# Chromatic Acclimation Processes and Their Relationships with Phycobiliprotein Complexes

**DOI:** 10.3390/microorganisms10081562

**Published:** 2022-08-03

**Authors:** Fanyue Wang, Min Chen

**Affiliations:** School of Life and Environmental Sciences, University of Sydney, Sydney, NSW 2006, Australia; fwan8133@uni.sydney.edu.au

**Keywords:** cyanobacterial photoreceptors, phycobiliproteins, chromatic acclimation, linker proteins, phylogenetic comparison

## Abstract

Chromatic acclimation (CA) is a widespread mechanism for optimizing the composition of phycobiliprotein complexes to maximize the cyanobacterial light capture efficiency. There are seven CA types, CA1-CA7, classified according to various photoregulatory pathways. Here, we use sequence analyses and bioinformatics to predict the presence of CA types according to three GAF (cGMP phosphodiesterase/adenylyl cyclase/FhlA)-containing photoreceptors, CcaS (cyanobacterial chromatic acclimation sensor), RcaE (regulator of chromatic adaptation), and RfpA (regulator for far-red photoacclimation). These photoreceptors were classified into three different phylogenetic groups leading different CA types in a diverse range of cyanobacteria. Combining with genomic information of phycobilisome compositions, the CA capabilities of various cyanobacteria were conjectured. Screening 65 accessible cyanobacterial genomes, we defined 19 cyanobacteria that have the capability to perform far-red light photoacclimation (FaRLiP) under the control of RfpA. Forty out of sixty-five cyanobacteria have the capability to perform green/red light photoacclimation, although they use different photoreceptors (RcaE and/or CcaS) and photoregulatory pathways. The reversible response of photoreceptors in CA regulation pathways trigged by changed light conditions reflects the flexibility of photoregulatory mechanisms in cyanobacteria and the putative independent evolutionary origin of photoacclimation types.

## 1. Introduction

Cyanobacteria are primary producers and contribute significantly to the global carbon and nitrogen flows [1]. They exist in diverse ecological niches ranging from desert to rock surface, ice sheet, saline soil, or forest soil, from fresh water to marine water bodies. As photoautotrophic organisms, light is an imperative energy source and an essential environmental indicator [2]. Since light can vary both in quality and quantity, sensing and responding to light are key mechanisms of cyanobacterial eco-physiological versatility, including increasing light-harvesting efficiency for photosynthesis.

The cyanobacterial photosynthetic apparatus comprises two major pigment-binding protein complexes: chlorophyll (Chl)-binding protein complexes localized inside the thylakoid membranes and bilin-binding protein complexes localized outside thylakoid membranes. Several different types of light-harvesting bilin-binding proteins are classified based on the bound chromophore types responsible for their different absorption maxima: phycocyanin (PC) bound phycocyanobilin (PCB) and gives the absorption maximum (λ_max_) = ~620 nm [3]; phycoerythrin (PE) bound phycoerythrobilin (PEB) and gives the λ_max_ = ~565 nm; phycoerythrocyanin (PEC) bound PCB or PVB and gives the λ_max_ = ~575 nm [3,4]; and allophycocyanin (APC) bound PCB and gives the red-shifted λ_max_ = ~650 nm [5]. Multiple bilin-binding proteins (also named as chromophorylated phycobiliproteins) form an α_6_β_6_ oligomers disc unit and those discs are assembled by their associated non-chromophorylated linker proteins and are integral to the supramolecular phycobilisome (PBS) complexes. PBS is the main antenna system for cyanobacteria and captures light of wavelengths that are poorly absorbed by Chls and transfers the energy to Chls in photosystems [6]. The organization of PBS structure relates to the presence of PBS linker proteins, especially the linkers associated with APC core and membranes [7]. The linker proteins facilitate the assembly of PBS and modulate the absorption properties of PBS, promoting the unidirectional down-hill energy transfer pathways from outside short-wavelength absorption rod structures (PEC, PE, and PC) to the core structure of APC, and then to photosystems supporting the light-harvesting efficiency [8].

In many cyanobacteria, the composition of PBS can change to accommodate the prevalent wavelength of light in the environment. Chromatic acclimation (CA) is a phenomenon observed in cyanobacteria, in which cyanobacteria perceive the wavelength of light in the environment and transduce the light signals into a sequence of biochemical events resulting in changing the activities of genes encoding specific phycobiliproteins [9,10]. Cellular CA responses represent the immediate changes occurring by changes at the level of gene expression. In addition, light intensity and nutrients can change gene expression profiles and PBS structures. The current article will focus on the diversity of molecular events involved in CA by screening known genomic information. Most of the information concerning green/red and red/far-red transducing reactions are presented here. 

Currently, seven CA variants have been classified and defined as CA1–CA7 [11,12]. The CA1, CA2, CA3, and CA7 represent the response to green and red light [13]; CA4 represents the response to green and blue light [14,15]; CA5 and CA6 govern photo-regulation triggered by red and far-red light. However, the photoreceptor regulating CA4 (Green/blue light acclimations) and CA5 (reported in *Acaryochloris marina* MBIC11017 only) are still to be defined [16,17]. Here, we studied the types of CA regulated by GAF (cGMP phosphodiesterase/adenylyl cyclase/FhlA)-containing photoreceptors. GAF domain-containing photoreceptors use bilins as their chromophore and, typically, can absorb light in two regions of the wavelengths to interchange between photoconvertible isomers [18]. The types of CA responding to green/red light are mainly regulated either by the chromatic acclimation sensor of CcaS reported in CA1, CA2 and CA7 or the chromatic acclimation sensor of RcaE governing CA3 processes [11,12,19]. The canonical CA3-capable cyanobacterium, *Fremyella diplosiphon*, has almost no PE in red light conditions and each rod contains three PC discs (hexamers). This organism will synthesize PE, and each rod contains two to three PE discs and one PC disc under green light conditions [20]. Both CcaS and RcaE photosensors contain the PAS/GAF core modules and defined as a green/red photosensor using different photo-regulatory pathways [21]. The GAF domain of CcaS has the maximal absorption at wavelengths of 536 nm (green) and 672 nm (red) [19]. CA1 shows no altered levels of red-light absorbing PC and green light-absorbing PE in the rod of PBS and a newly assembled CpcL-rod-shaped PBS alternatively associated with Photosystem I under prevalent green light conditions [22,23]. CcaS leads to green-activated phosphorylation of transcriptional regulator CcaR to regulate the expression of a gene operon that encodes the rod–membrane linker (CpcL) in CA1, PE-associated rod linkers and regulators in CA2, and PEC in CA7 [11]. CA2 is controlled by the CcaS photoregulatory pathway and exhibited that PE levels are photo-acclimated components in response to the changed light signals [19,24]. 

CA3 is also known as complementary chromatic acclimation (CCA), which showed that PE is accumulated under green light and PC differentially accumulates in the outer portion of PBS rods under red light [13]. RcaE transduces the light signals through two response regulators, RcaF and RcaC, to control the pigment content of PBS associated with CA3 responses. In red light, genes encoding PE proteins are repressed and in the green light, the repression of PE synthesis genes are removed resulting in PE accumulation [2,21]. 

The newly defined CA6 regulates the far-red-absorbing variants of PBSs to couple with far-red light photo-acclimation (FaRLiP) upregulation and Chl *f* production [25,26,27,28]. The FaRLiP gene cluster is controlled by a three-gene operon, *rpfB*-*rfpA*-*rpfC*, and protein RfpA is a phytochrome photoreceptor containing a PAS, GAF and histidine kinase domains [27,29]. The null mutant of three genes, *rfpA*, *rfpB,* and *rfpc*, caused no CA6-capability in CA6-capable cyanobacteria [27]. Here, we analyzed publicly available cyanobacterial genomes and show that 19 cyanobacteria have the FaRLiP gene cluster containing a three-gene operon, *rpfB*-*rfpA*-*rpfC*. 

CcaS, RcaE and RfpA have a common bilin-binding GAF domain and have a unique photocycle in response to the changed illumination [27,30]. However, the CcaSR, RcaEFC and RfpABC photosensory systems show distinct signal transduction pathways and target different PBS genes, suggesting that they originate from a common photosensory system and differentiated during the evolution of CA in cyanobacteria. PBS proteins are a major constituent in the cells that allows cells to optimize absorption of light for photosynthesis. Most CA-capable cyanobacteria are able to assemble two different PBSs based on their light wavelength-dependent accumulation of phycobiliproteins and their associated linkers. The cooperation of the variety of PBS becomes an essential element for multiple photoregulatory pathways to enhance the efficiency of photoacclimation and provide cell growth advantage. Here, we report that CA types controlled by CcaS, RcaE or RfpA are widespread throughout the cyanobacterial phylum. Comparing conserved C-terminal protein sequences of CpcG (PBS rod–core linker protein) and CpcL (PBS rod–membrane linker protein), we reannotated CpcL encoding genes and the possibility of the formation of CpcL-rod PBS induced by corresponding light signals was predicted, which may have direct implications on the regulation of the structure of PBS in vivo. 

## 2. Materials and Methods

### 2.1. Selected Cyanobacteria Genomes and Predicated Photosensory Proteins 

The representatives’ species covering each defined CA type were selected after retrieving GAF-containing photoreceptors of CcaS, RcaE, and RfpA, and the characteristic PBS from the accessible genomic datasets. The selected sixty-five cyanobacterial genomic sequences were obtained from GeneBank (NCBI) and Integrated Microbial Genomes of the DOE joint Genome institute (Table S1). 

16S rRNA sequences were deduced from the genome sequence and used to classify cyanobacteria into groups (Table S1). Further cyanobacterial taxonomy information was retrieved from JGI IMG database (Joint Genome Institute (JGI) Integrated Microbial Genomes (IMG); https://img.jgi.doe.gov/, accessed on November 2021–May 2022).

An array of GAF-containing protein homologs was defined after genomic annotation and re-defined by the BLASTP program at www.ncbi.nlm.nih.gov to screen for the presence of putative GAF-containing proteins. The conserved protein domains and domain structure of predicted GAF-containing proteins were compared and reannotated after the known photoreceptors reported from cyanobacteria, such as CcaS of *Synechocystis* sp. PCC 6803 (WP_014407164) and RcaE of *Fremyella diplosiphon* Fd33 (WP_045871568). RfpA homologs were determined after defined FaRLiP cluster and known RfpA protein module organization of *Halomicronema hongdechloris* C2206 (WP_080806407). 

Domain organization of photoreceptor proteins was determined using the Conserved Domains Search program in NCBI connected with the Pfam (protein families) database. The CA types were predicted by combining the presence of photoreceptors and photo-acclimated PBS components based on the definition of CA types [17].

### 2.2. Sequence Homology and Phylogenetic Analysis

The sequences of GAF domains (lengths of 149–174 amino acids) from representatives of CcaS, RcaE and RfpA homologs were extracted after defined functional domains. The multiple sequence alignments were generated through the MEGA-5 program using ClustalW program [31] and refined after published GAF-domain alignment in Hirose et al. [30]. The phylogenetic analyses were performed using the Neighbour-Joining method and Jones–Thornton–Taylor (JTT)-based model in MEGA-5 [31]. The equal Aa substitution rates were used, and the consensus trees were generated and supported by 5000 bootstrap replicates.

Protein sequences of phycobiliproteins and linker proteins were obtained using BLASTP searches through the selected cyanobacterial genomic sequences to collect any possible mis-annotated linker proteins. The PBS rod–core linker proteins (CpcGs, containing PBS linker domain Pfam00427) were verified and separated CpcL (also containing PBS linker domain Pfam00427) from CpcG linkers by comparing the predicted secondary structure if there was a hydrophobic helix at the C-terminal region (https://services.healthtech.dtu.dk/service.php?TMHMM-2.0, accessed on March–June 2022). The phylogenetic analyses of linker proteins were performed using the Neighbour-Joining method and Jones–Thornton–Taylor (JTT)-based model in MEGA-5 [31]. The equal Aa substitution rates were used, and the consensus trees were generated and supported by 500 bootstrap replicates.

The genes encoding 15,16-dihydrobiliverdin:ferredoxin oxidoreductase (PebA) and phycoerythrobilin:ferredoxin oxidoreductase (PebB) or their homologs are used to verify the presence of PEB-associated protein subunits, PE-rods. 

## 3. Results and Discussion

### 3.1. Photoreceptors and CA-Capable Cyanobacteria

#### 3.1.1. Predication of Photoreceptors

**Figure 1:**
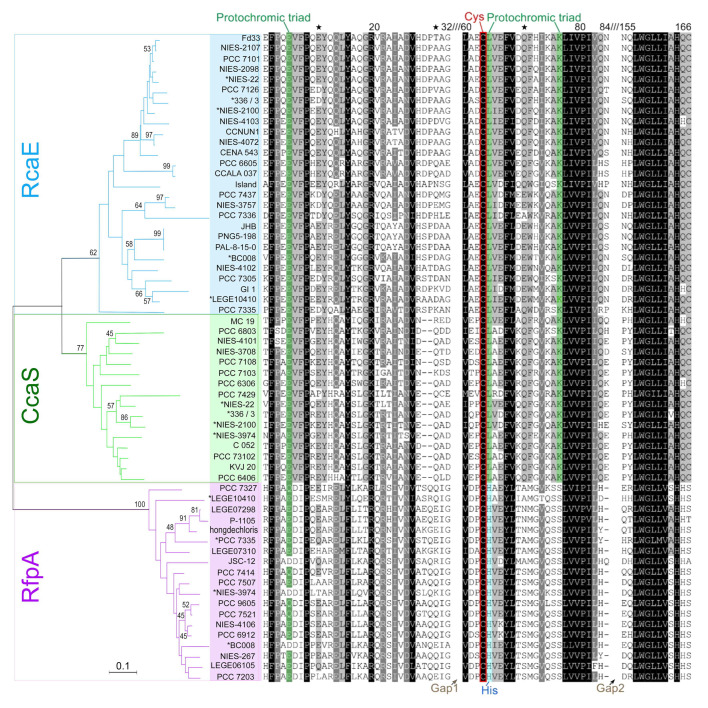
Phylogenetic analysis and GAF domain alignments of photoreceptors. The groups of RcaE (Blue), CcaS (green), and RfpA (purple) are supported by the alignment fragments of GAF domains. The conserved Cys sites are highlighted in a red framed box. The protochromic triad sites are highlighted in green and conserved His sites of RfpA are highlighted in blue. The asterisk represents that the strain uses two different photoreceptors. The branch supports are calculated by 5000 replicates. The five-pointed stars represent the positions of Aa alignment at 10, 30, and 70, respectively. The strain culture numbers are used in the figure and the photoreceptor sequence accessions are listed in Table S1.

The GAF-containing protein homologs were retrieved after screening 65 selected cyanobacterial genomes. All retrieved GAF-containing proteins were classified by comparing the protein domain architectural structure to the characterized CcaS (WP_014407164), RcaE (WP_045871568), and RfpA (WP_080806407) (Figure S1). The constructed phylogenetic tree demonstrated three photoreceptor groups, verifying the prediction of photoreceptors (Figure 1). The chromophore binding sites (Cys) are conserved in three photoreceptors. Different amino acids involved in the function of the protochromic triad are noticed in RfpA. Conserved histidine (His) next to Cys is consistent in the GAF of RfpA and replaced leucine (Leu) in the CcaS and RcaE (Figure 1). The imidazole group of His represses the thiol adduct formation and plays an important role for the cleaving of the thioether linkage in the 15E state of bilins in RfpA, supporting red/far-red conversion [32]. 

#### 3.1.2. Characterization of CA-Capabilities 

**Figure 2:**
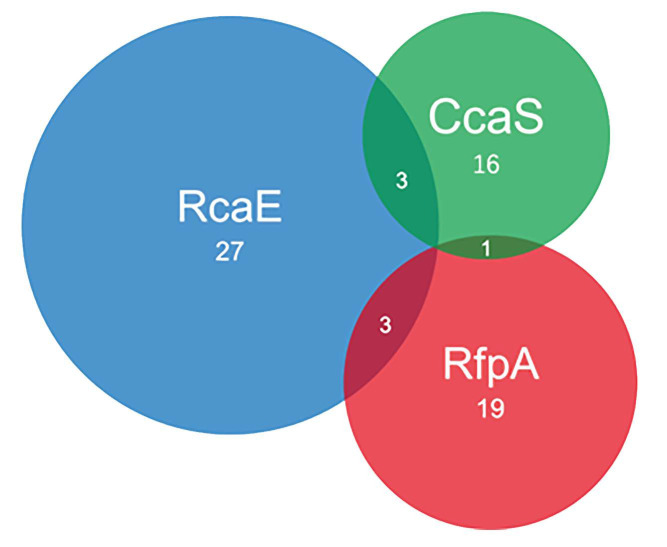
Venn diagram summarized the numbers of photoreceptors from 65 selected cyanobacterial genomes. The numbers in the overlayed regions represent the cases of co-existing photoreceptors. Green, CcaS; Blue, RcaE; Red, RfpA.

After screening the 65 cyanobacterial genomes, 16 out of 65 cyanobacteria contain CcaS homologs of *Synechocystis* sp. PCC 6803, including 3 of them that also contain the RcaE photoreceptor (Figure 2). The co-existing CcaS and RcaE in *Calothrix* sp. NIES-2100, Calothrix brevissima NIES-22, and *Calothrix* sp. 336/3 allow them to conduct multiple types of green-/red-light-induced CA, including CA1, CA2, and CA3 (Figure 2; Table 1). 

**Figure 3:**
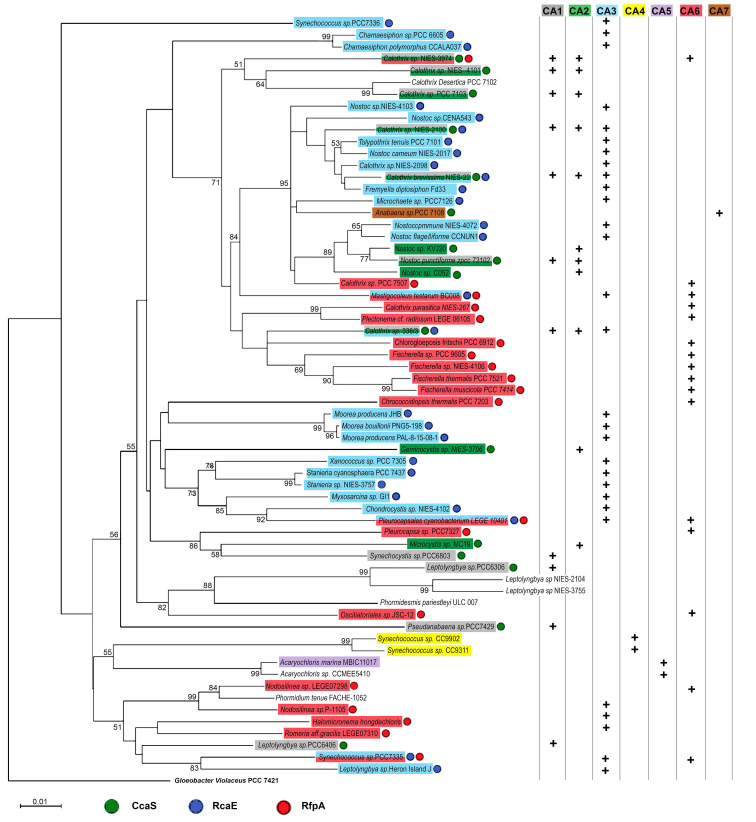
Neighbor-joining phylogeny of selected 65 cyanobacteria based on 16S rRNA sequences and defined chromatic acclimation (CA) types. The CA types are color-coded as CA1, gray; CA2, green; CA3, blue; CA4, yellow; CA5, purple; CA6, red; and CA7, brown. The photoreceptors are illustrated as circles next to the cyanobacterial names and are color-coded as CcaS (Green), RcaE (Blue), and RfpA (Red). The branched groups are supported with a bootstrap of ≥50%. The scale bar represents a phylogenetic distance of 0.01 nucleotide substitutions per site. All sequence accessions are listed in Table S1.

Twenty-seven out of 65 cyanobacteria contain RcaE homologs of *F. diplosiphon*, and all of them have the capability to synthesize PEB (Table S1; Figure 2). The occurrence of the RcaE photoreceptor is directly related to the capability of assembling PE-containing PBS and it is independent of cyanobacterial families (Figure 3).

**Figure 4:**
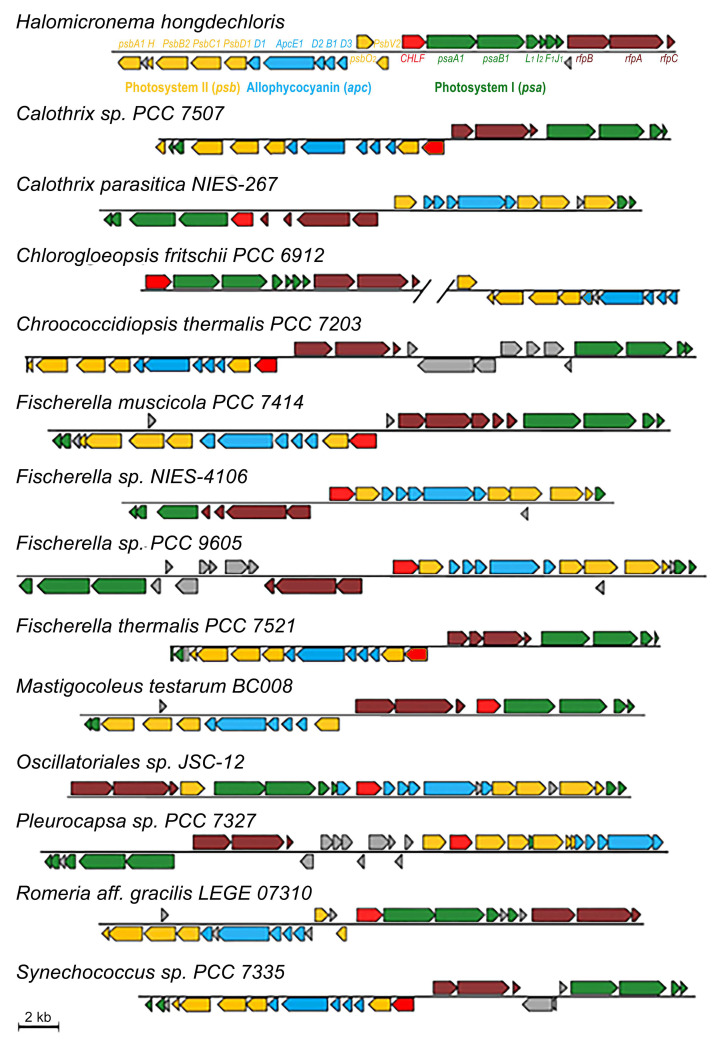
FaRLiP gene clusters from 14 CA6-capable cyanobacteria. The identities of the genes from *H. hongdechloris* are used as references. Blue represents the Allophycocyanin (Apc) subunits; green represents genes encoding photosystem I (*psa*); yellow represents genes encoding photosystem II (*psb*); red represents Chl *f* synthase (CHLF) homolog; dark-red represents the Rfp operons (*rfp*ABC); gray represents hypothetical proteins.

Initial searches for RfpA homologs (WP_080806407, *H. hongdechloris*) were performed via BLASTP and returned many hits of GAF domain-containing proteins. We combined the results of searching for the FaRLiP cluster near the putative RfpA homologs or operon of *rfpB*-*rfpA*-*rfpC* and defined 19 out of 65 cyanobacteria containing the FaRLiP gene cluster including 5 cyanobacteria their FaRLiP cluster placed in different genomic scaffolds (Figure 4 and Appendix S1). As demonstrated in Figure 4, all 14 FaRLiP clusters contain core subunits of PSI, PSII and PBS except *Fischerella* sp. NIES-4106. *Fischerella* sp. NIES-4106 contain only one copy of *psaA* but two copies of *psaB*. The PsaB encoding gene in the FaRLiP cluster of *Fischerella* sp. NIES-4106 is shorter, ~602 Aa, due to missing ~150 Aa at the N-terminal of typical PsaB. A different organisation of photosynthetic apparatus induced by CA6 could be expected in *Fischerella* sp. NIES-4106 due to dysfunctional PSI core encoding genes in FaRLiP cluster. The presences *rfpBAC* operon and FaRLiP cluster confirmed they are CA6-capable cyanobacteria. 

There are RfpA-like homologs detected in *Calothrix desertica* PCC 7102 and *Nostoc sp.* C052, but no detectable FaRLiP gene cluster in the genomes (data not shown). Those RfpA-like photoreceptors are not involved in CA6-type photoacclimation and their regulatory functions are unknown. Interestingly, screening the genomic draft of *Calothrix desertica* PCC 7102, we could not find homologs of CcaS and RcaE, although there are 27 GAF-containing hypothetical proteins (Table S1). We proposed that *Calothrix desertica* PCC 7102 does not have known CA capability. Additionally, another five out of 65 cyanobacteria demonstrated no known CA1-CA3 and CA6 capability due to lacking known CA-regulators of CcaS, RcaE, or RfpA including the outgroup cyanobacterium *Gloeobacter violaceus* PCC 7421. One common feature of those non-CA capable cyanobacteria, excluding the outgroup of *Gloeobacter violaceus* PCC 7421, is that APC and PC are dominant subunits of PBS and no PE and PE-associated encoding genes are found in their genomes (Table S1). However, *Acaryochloris* sp. CCMEE 5410 is assigned as non-CA capable cyanobacterium due to lacking PBS rod encoding genes. 

As described above, the altered ratio of PE:PC in PBS rods reflects the spectral distribution of green/red light and is governed mainly by photoreceptors of CcaS and RcaE. In this report, 26 out of 65 cyanobacteria do not have PE proteins, including 1 CA7 representative, 2 CA5-capable representatives, and 4 CA1-capable cyanobacteria (Table S1). 

Most CA6-capable cyanobacteria possess PBS without involvement of PE and only 5 out of 19 CA6-capable cyanobacteria contain PE encoding genes. The far-red light photosensor of RfpA co-exists with RcaE in *Mastigocoleus testarum* BC008, *Pleurocapsales cyanobacterium* LEGE 10410, and *Synechococcus* sp. PCC 7335, resulting in their complicated photoacclimation response of green/red (CA3) and red/far-red (CA6). The three CA3 and CA6 enabling cyanobacteria belong to three different orders of cyanobacteria and have different morphological features, suggesting the CA development is independent of the morphological properties and their family orders (Table 1). In *Synechococcus* sp. PCC 7335, a null mutant of *rfpA* affected the ratios of PE:PC, suggesting the interaction between CA3 and CA6 regulatory pathways [33]. 

### 3.2. Cyanobacterial PBS Structure and CA Responses 

Tuning PBS composition in response to distinct wavelengths has been recognised, such as the PBS having extended PE rods will prefer to capture green light observed in CA2-capable cyanobacteria. Knowing the genes encoding phycobiliproteins and linkers by screening genomic information could help us to predict the involvement of PBS in CA processes. In this section, we identified the possible types of PBS according to the PBS encoding gene profiles.

#### 3.2.1. Changeable PE Rods

The presence of PE encoding genes was learned from published genomic sequences and confirmed by capability of PEB synthesis, i.e., if there are PebA and PebB encoding genes annotated in the genome. In total, 39 out of 65 cyanobacteria can synthesize PE and only two cyanobacteria, *Calothrix desertica* PCC 7102 and *Gloeobacter violaceus* PCC 7421, demonstrate constitutively assembling PE-containing PBS without predicted photoacclimation changes. The assigned 27 CA3-capable cyanobacteria will change the ratio of PE:PC in the rod under control of the RcaE mechanism (Table S1). The altered ratio of PE:PC results in visible cellular color changes. No PE component can be detected in the CA3-capable cyanobacteria grown under red light conditions, resulting in blue-green colored appearance [13].

The assigned 11 CA2-capable cyanobacteria contain the CcaS regulatory pathway. The upregulating product of PE in green light extended the rod length without changes of PC amount at the base part of the rod [12]. PE-associated genes are constitutively active and PE subunits are an important composition of PBS independent of culture conditions, but PE components will be increased in green light conditions. 

PE is the main acclimating rod component controlled by green light in CA2- or CA3-capable cyanobacteria. However, not all PE-containing PBS photoacclimate. Approximately 27% of PE-containing cyanobacteria were not capable of altering either PE or PC amount under changing light colors [13]. The photoacclimating morphological changes are only observed in CA2- and CA3-capable cyanobacteria (Table S1). For example, *Gloeobacter violaceus* PCC 7421 demonstrated purple cellular color due to constitutive expression of PE encoded genes, which are not governed by photoacclimation. 

#### 3.2.2. Assembling CpcL-Rod PBS Structure

**Figure 5:**
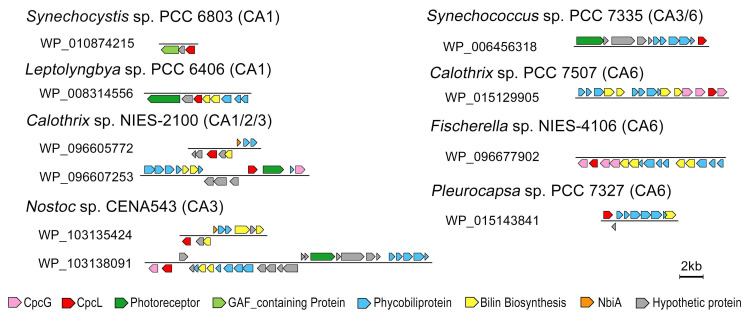
Associated gene locus containing *cpcL* from representative cyanobacteria.

CA1-capable cyanobacteria share the same photoreceptor of CcaS, although the gene organization controlled by CcaS might be differentiated to regulate products of PE or CpcL. The co-presence of the rod–membrane linker (CpcL) and CcaS regulatory pathways are the essential features of CA1-capable cyanobacteria [11]. In the process of CA1, CcaS mediate the product of *cpcL* gene and assemble phycobiliprotein rod interacting with PSI via CpcL to enhance energy distribution into PSI under green light conditions [34]. Both CpcL and CpcG contain a conserved linker domain of pfam00427 but have different hydrophilic/hydrophobic properties (Figure S2). CpcL serves as a rod–membrane linker and the hydrophobic helix at the C-terminal region enables it to connect the rod directly to photosynthetic reaction centers inside membranes [23]. In this study, we screened all annotated *cpcG* genes in the selective cyanobacterial genomes and predicted the presence of hydrophobic helices using the TMHMM program. In total, 42 out of 65 cyanobacteria contain CpcL encoding genes, while only 11 of them contain photoreceptor CcaS. Reading through the locus of *cpcL*, we noticed that most *cpcL* genes are localized in one or another phycobiliprotein associated operons (Figure 5). The formation of CpcL-rod PBS could be regulated together with other phycobiliproteins by sharing photoreceptors and photo-regulatory pathways. 

As shown in Figure 5, *cpcL* is localized in the operon containing photoreceptors or close to the gene encoding phycobilin lyase (such as *cpcS*, *cpcE*, and *cpcF*) and phycobiliproteins. Assembling simple CpcL-rod PBS appears to be a commonly used mechanism to balance the energy flow between PSI and PSII, especially under certain light and nutrient conditions [35]. The CpcL-rod PBS was only isolated from green light cultured CA1-capable cyanobacterium *Leptolyngbya* sp. PCC 6406, indicating that the expression level of *cpcL* was regulated by green light [11].

Previous reports also indicated that CpcL-rod PBS in some cyanobacteria are consistently present. In CA5-capable cyanobacterium *A. marina* MBIC11017, CpcL-PC-rod-shaped PBS is the basic PBS structure associated with PSI and PSII [36]. In *Anabaena* sp. PCC 7120, isolated PBS-CpcL-PSI complexes indicated the constant expression of the CpcL encoding gene without involvement of photoregulatory mechanisms [35].

Within the 19 identified CA6-capable cyanobacteria, only three of them including *H. hongdechloris* do not have CpcL encoding genes (Table S1). Most *cpcL* genes from CA6-capable cyanobacteria are localized within a phycobiliprotein operon containing phycobiliprotein subunits and phycobilin assembling/biosynthesis enzymes (Figure 5). Interestingly, phylogenetic analysis of CpcL revealed a separate group of CpcL from some CA6-capable cyanobacteria, although there are CpcLs from other CA6-capable cyanobacteria that are placed in different phylogenetic groups. Independent evolutionary/development pathways of CpcL-rod PBS and photoacclimation are proposed (Figure S3). 

There are 14 out of 27 CA3-capable cyanobacteria having CpcL encoding genes, and most *cpcL* genes are in the gene operon of phycobiliproteins modulated by RcaE or close to phycobilin biosynthesis enzymes, which might be modulated by different photoreceptors. Further investigation on the formation of CpcL-rod in CA3-capable cyanobacteria could reveal updated knowledge of photoacclimation and adaptive photosynthetic apparatus. 

#### 3.2.3. PBS Having Red-Shifted Absorption Characteristics

There are two different PBS structures supported by two copies of core–membrane linker encoding genes (*apcE*) in CA6-capable cyanobacteria (Table S1) [28]. The conventional PBS are assembled under white light conditions, and red-shifted PBS are formed under far-red light conditions. All CA6-capable cyanobacteria have five additional genes encoding APC subunits in the FaRLiP gene cluster, which are essential APC subunits assembling the PBS core with red-shifted absorption [28]. The five APC encoding genes include three ApcD, one ApcB and one ApcE (Figure 4). 

ApcE is a high molecular mass core–membrane linker and has a homology to phycobiliproteins at the N-terminal region and several repeats of linker polypeptides at the C-terminal region [6]. The phycobiliprotein-like N-terminal domain can bind a PCB and serve as a PBS terminal energy acceptor [6]. The number of repeats of linker domains of ApcE are different according to the size of assembled PBS [28]. In CA6-capable cyanobacteria, the product of *apcE* in the FaRLiP gene cluster has two repeats of linker domain, a shorter version of ApcE, assembling a smaller size of PBS. The smallest PBS isolated from far-red light grown *H. hongdechloris* culture provided experimental evidence that ApcE from FaRLiP is involved in assembling small PBS and modulates the absorption properties [25]. Figure 4 demonstrates that five gene clusters of *apcD-E-D-B-D* are present in all CA6-capable cyanobacteria and only *Oscillatoriales* sp. JSC-12 has an additional ApcD encoded gene in the cluster (Figure 4). These APC subunits are responsible for the red-shifted absorption of PBS isolated from *H. hongdechloris* [25,28]. The disorganized structure of PBS due to newly synthesized APC subunits induced by CA6 contributed to the changed spectral properties of PBS in *Synechococcus* sp. PCC 7335 [37]. 

**Figure 6:**
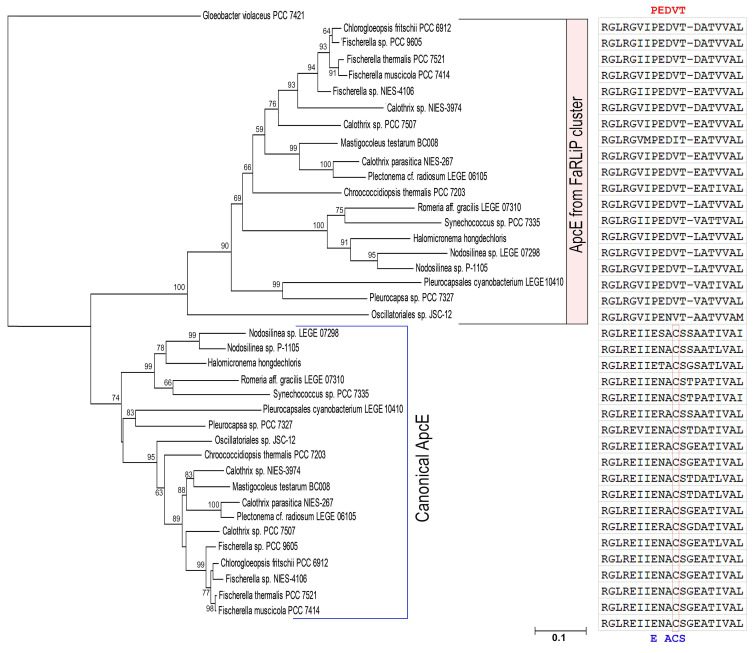
Neighbor-Joining phylogeny and alignment of conserved phycobiliprotein region of ApcEs from CA6-capable cyanobacteria. The conserved Cys sites from canonical ApcE proteins are highlighted in a red framed box. The conserved motif PEDVT in ApcE from FaRLiP cluster are highlighted in red. The conserved motif ExACS in canonical ApcE are highlighted in blue. The ApcE sequence accessions are listed in Table S1.

ApcE proteins from FaRLiP not only have fewer repeats of linker domain, but also lack chromophore-binding sites (conserved cysteine, Cys) in the phycobiliprotein-like domain (Figure 6) [25,33]. Using the N-terminal domain of ApcE, we can separate canonical ApcE from the ApcE in the FaRLiP cluster (Figure 6). Additionally, one ApcD from FaRLiP also lacks chromophore binding sites [38]. The missed chromophore binding sites of phycobiliproteins/domains might play a vital role in modulating the spectral properties of PBS induced by CA6 [37,39]. 

### 3.3. Photoacclimation and Alternative PBS

The hemidiscoidal shape of PBS is the typical form, which consists of a central APC core and radiating either PC or PC and PE/PEC rods. With variability in the rod length across species (e.g., CA3-capable cyanobacteria), re-equilibrated energy distribution among photosystems (e.g., CA1-capable cyanobacteria), and inducible PBS consisting of only the core component (e.g., CA6-capable cyanobacteria), altered PBS morphologies reflect the multi-CA-type and photoregulatory pathways. Sixty-five cyanobacteria were selected covering variations of PBS and CA types. Our results offer new insights into how cyanobacteria can flexibly combine different types of CA to acclimate to different light environments. However, with extensively increased numbers of fully sequenced cyanobacteria, new studies on photoreceptors in caynobacteria demonstrated that CA are also modulated by multiple photoreceptors. For example, cyanobacteriochromes (CBCRs) can sense a wide range of wavelengths and modulate CA by refined wavelength resulting in more complicated dual or triple control of CA processes [18,40]. In 2016, Wiltbank and Kehoe demonstrated a photoreceptor DpxA (decreased phycoerythrin expression A) that senses different wavelengths of light from RcaE and regulated the amount of PE in CA3-capable cyanobacteria [41]. The proposed dual control system in CA3-capable cyanobacteria enables the organism to sense a wide spectrum of light with ‘fine-tuning’ CA responsive processes. A wide range of photoreceptors provide cyanobacteria flexibility in tuning cellular responses to light. Beyond highlighted photoreceptors retrieved from genomes and their functional role in photosynthesis, a range of GAF-containing proteins are involved in important cellular processes including growth, phototaxis, and cell aggregation, most of them are awaiting characterization and beyond current report scopes (Table S1) [18,42]. Here, we used photoreceptors, CcaS, RcaE and RfpA, that are known to regulate at least one CA process and predicted the similar physiological responses across cyanobacteria. Further studies on photoacclimation in cyanobacteria represent new research frontiers in understanding ‘switchable’ photosynthetic apparatus in cyanobacteria, but also in building new biotechnological tools. 

## 4. Conclusions

CA is a photoacclimation strategy widely distributed in cyanobacteria. In this study, 65 selected cyanobacterial genomes were explored for the relationship between CA photo-regulatory pathways and PBS components. Since the PBS compositions are the main switchable parts in response to changed light conditions, CA capabilities of cyanobacteria rely on the genetic features of PBS. We define the CA types according to the presence of known photoreceptors and potential components for assembling functional PBS. 

The phylogeny of conserved GAF domains from different photoreceptors verified the classifications of CcaS, RcaE, and RfpA. The different amino acid residues surrounding the bound chromophore sites indicate the different characterized active wavelength ranges of photoreceptors [26]. The conserved His next to Cys in RfpA play an important role for the active range of red/far-red wavelength [32]. Most CA-capable cyanobacteria can assemble two different types of PBS: (1) the different length of rod and ratio of PE:PC in CA2- and CA3-capable cyanobacteria; (2) co-existing canonical PBS structure serving for PSII and single rod-shaped PBS serving for PSI via CpcL linkers; (3) newly assembled, red-shifted PBS will replace the canonical PBS in CA6-capable cyanobacteria induced by far-red light. With detailed protein alignments, we predicted key motifs and conserved amino acid residues contributing to the spectral characteristics of photoreceptors and the shifted absorption of PBS. The different phylogenetic relationships based on 16S rRNA and photoreceptors suggest that cyanobacteria acquired the CA genes by horizontal gene transfer. However, an independent evolutionary origin of photoacclimation types does not directly reflect the adverse environments. 

## Figures and Tables

**Table 1:** Co-existing photoreceptors and chromatic acclimation types.

Cyanobacteria	Photoreceptors	CA Types				Phycobiliproteins	Linker Proteins
*Calothrix* sp. NIES-3974	CcaS/RfpA	CA1	CA2		CA6	APC/PC/PE	ApcE×2/CpcG×3/CpcL×2
*Calothrix* sp. NIES-2100	CcaS/RcaE	CA1	CA2	CA3		APC/PC/PE	ApcE×1/CpcG×1/CpcL×2
*Calothrix brevissima* NIES-22	CcaS/RcaE	CA1	CA2	CA3		APC/PC/PE	ApcE×1/CpcG×2/CpcL×2
*Calothrix* sp. 336/3	CcaS/RcaE	CA1	CA2	CA3		APC/PC/PE	ApcE×1/CpcG×2/CpcL×2
*Calothrix* sp. 336/3	CcaS/RcaE	CA1	CA2	CA3		APC/PC/PE	ApcE×1/CpcG×2/CpcL×2
*Mastigocoleus testarum* BC008	RcaE/RfpA			CA3	CA6	APC/PC/PE	ApcE×3/CpcG×1/CpcL×2
*Pleurocapsales cyanobacterium* LEGE 10410	RcaE/RfpA			CA3	CA6	APC/PC/PE	ApcE×2/CpcG×1
*Synechococcus* sp. PCC 7335	RcaE/RfpA			CA3	CA6	APC/PC/PE	ApcE×2/CpcG×1/CpcL×1

## Data Availability

Accession numbers and publicly archived datasets used in this study can be found in Supplementary Table S1.

## Supplementary Materials

The following supporting information can be downloaded at: https://www.mdpi.com/article/10.3390/microorganisms10081562/s1, Figure S1: The domain architecture of known photoreceptors (CcaS, RcaE, and RfpA); Figure S2: The differences between CpcG and CpcL; Figure S3: Neighbor-joining phylogeny of CpcL proteins; Table S1: Genetic information of selected 65 cyanobacteria; Appendix S1: Prediction of FaRLiP clusters retrieved form incomplete genomes (containing multiple scaffolds).
