# Supplementary material for: Chromatic Acclimation Processes and Their Relationships with Phycobiliprotein Complexes
Source: Microorganisms. 2022 Aug 3;10(8):1562. doi: 10.3390/microorganisms10081562 (PMC9415938; doi:10.3390/microorganisms10081562)
Supplement: Supplementary file 1 [file microorganisms-10-01562-s001.zip › microorganisms-1828046-supplementary.pdf]

Appendix S1

FaRLiP cluster information from five incomplete genome drafts

1. *Calothrix* sp. NIES-3974 incomplete genome draft

Scaffold *Calothrix sp.* NIES-3974 : Ga0263577_11 (5985875bp) contains small photosystem I subunits of PsaJ and PasF, Potosystem II subunits, allophycocyanin subunits and *rfpBAC* operon. The genes *psaA*/*psaB* are placed in a different position.

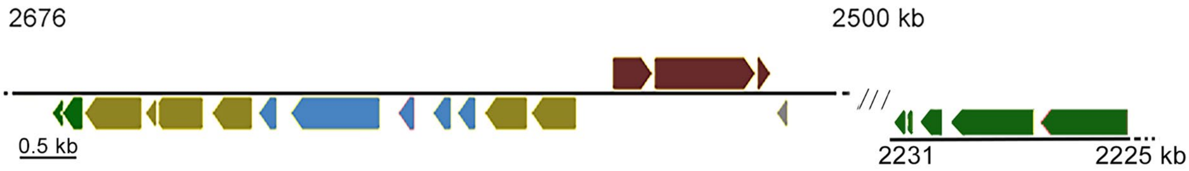

2. *Nodosilinea* sp. LEGE 07298 incomplete genome draft

Scaffold Ga0481345_168 : *Nodosilinea* sp. LEGE 07298 (13672bp) contains psaA/psaB and allophycocyanin of ApcD and ApcB, partial ApcE. Scaffold Ga0481345_258 : *Nodosilinea* sp. LEGE 07298 (9293bp) start from partial ApcE encoding gene that next to ApcD. Then photosystem II encoded genes. However, with short scaffolds, we could not map the position of *rfpBAC* operon associated with the partial FaRLiP cluster.

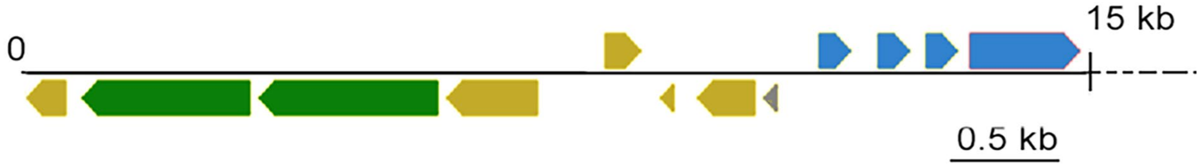

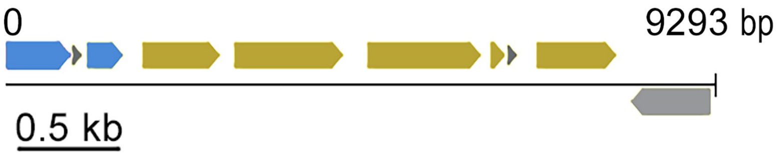

3. *Nodosilinea* sp. P-1105 incomplete genome draft

Five Allophycocyanin encoding genes are clustered with photosystem II gene in Scaffold: Ga0442552_125 : *Nodosilinea sp.* P-1105 (55594bp). One PsaA (without paired psaB gene) encoded gene at the end of this scaffold. There are three annotated *psaA*/*psaB* genes and 2 pairs of *psaA*/*psaB* found in the scaffolds. The third *psaB* gene (not paired with *psaA*) is localised in scaffold Ga0442552_194 : *Nodosilinea* sp. P-1105 (14349bp), containing *rfpBAC* operon.

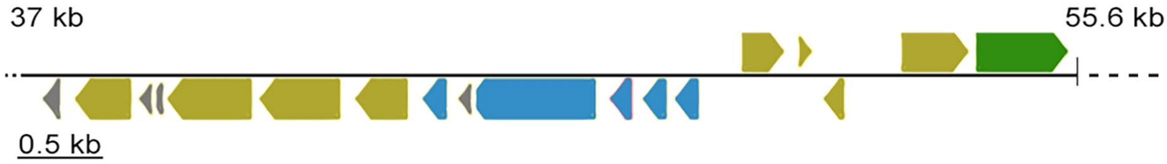

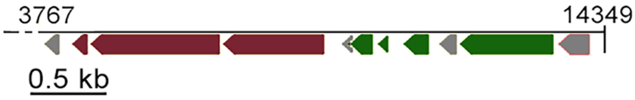

4. *Plectonema cf. radiosum* LEGE 06105 incomplete genome draft

Scaffold Ga0481330_094 : *Plectonema c f. radiosum* LEGE 06105 (29067bp) contains *rfpBAC* operon, allophycocyanin subunits and photosystem II encoded genes. Additionally, 2 PsaA and PsaB encoded genes in two different scaffold supports the presentence of FaRLiP cluster.

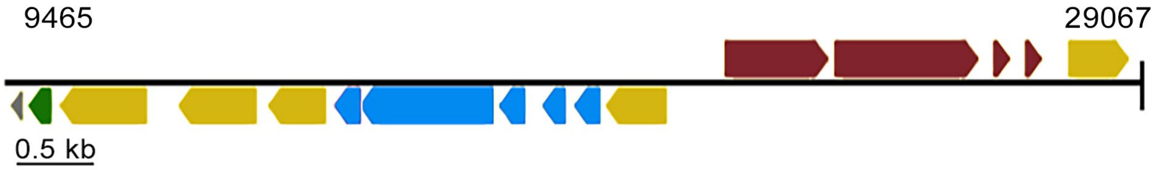

5. *Pleurocapsales cyanobacterium* LEGE 10410 incomplete genome draft

Scaffold Ga0481350_028 : *Pleurocapsales cyanobacterium* LEGE 10410 (20487bp) contains allophycocyanin subunits and photosystem II encoded genes. 2 copies of *psaA* /*psaB*, in which a pair of *psaA*/*psaB* are next to the predicted *rfpBAC* operon in scaffold Ga0481350_034 : *Pleurocapsales cyanobacterium* LEGE 10410 (20063bp).

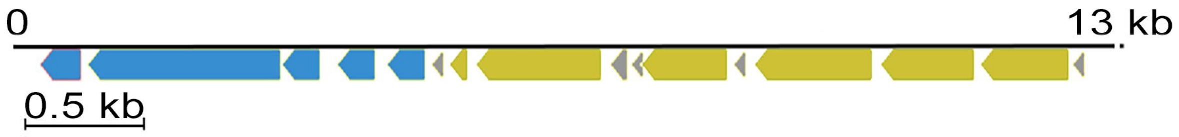

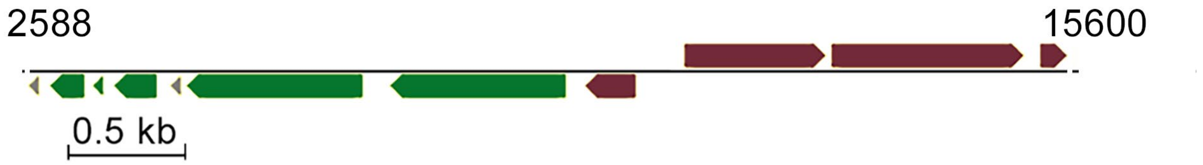

*Colour codes: Photosystem I, Photosystem II, Allophycocyanin, rfpBAC operon, and hypothetic proteins.

Supplementary Table S1. Genetic information of selected 65 cyanobacteria

Cyanobacteria	CA types	*Photoreceptor /the numbers of GAF-containing proteins	Chlorophylls	Phycobili-proteins	Bilin chromophores	Core-Linker proteins (x numbers)	Taxonomy (order/family)	Genome Accession	Photoreceptors accessions	ApcE Accession (CA6 cyanobacteria)	CpcG Accessions	CpcL Accessions
Acaryochloris marina MBIC11017	CA5	N.F./ 13	*a, d*	APC/ PC	PCB	CpcL×3	Synechococcales/ Acaryochloridaceae	CP000843, CP000839, CP000841, CP000846, CP000844, CP000842, CP000838, CP000840, CP000828, CP000845				AM1_C0092, AM1_C0102, AM1_C0203
Acaryochloris sp. CCMEE 5410	N.F	N.F./17	*a, d*	APC	PCB	N.F.	Synechococcales/ Acaryochloridaceae	AFEJ00000000				
Anabaena sp. PCC 7108	CA7	CcaS /15	*a*	APC/ PC	PCB	ApcE×1/ CpcG×3	Nostocales/ Nostocaceae	AJWF00000000	WP_016952251		WP_016950360, WP_016950361, WP_016950362	
Calothrix brevissima NIES-22	CA1/ CA2/ CA3	CcaS + RcaE/37	*a*	APC/ PC/ PE	PCB/ PEB	ApcE×1/ CpcG×2/ CpcL×2	Nostocales/ Calotrichaceae	AP018215, AP018213, AP018207, AP018211, AP018209, AP018214, AP018212, AP018210, AP018208	WP_096645556/ WP_096646471		WP_096645558, WP_096646466	WP_096645555, WP_096645004
Calothrix desertica PCC 7102	N.F.[b]	N.F./27	*a*	APC/ PC	PCB	ApcE×1/ CpcG×1/ CpcL×1	Nostocales/ Calotrichaceae	NZ_VLKB00000000			WP_127082892	WP_127082891
Calothrix parasitica NIES-267	CA6	RfpA /22	*a, f*	APC/ PC	PCB	ApcE×2/ CpcG×3/ CpcL×1	Nostocales/ Calotrichaceae	AP018230, AP018232, AP018228, AP018227, AP018229, AP018231	WP_096658604	WP_096658610, WP_096655631	WP_096654956, WP_096654958, WP_096654960	WP_096661134
Calothrix sp. 336/3	CA1/ CA2/ CA3	CcaS + RcaE/17	*a*	APC/ PC/ PE	PCB/ PEB	ApcE×1/ CpcG×2/ CpcL×2	Nostocales/ Calotrichaceae	CP011383, CP011385, CP011384, CP011382	WP_082127326/ WP_2338178504		WP_035159010, WP_035153937	WP_035153901, WP_035153933
Calothrix sp. NIES-2098	CA3	RcaE / 31	*a*	APC/ PC/ PE	PCB/ PEB	ApcE×1/ CpcG×2/ CpcL×1	Nostocales/ Calotrichaceae	AP018173, AP018172	WP_096589648		WP_096588437, WP_096589657	WP_096588435
Calothrix sp. NIES-2100	CA1/ CA2/ CA3	CcaS + RcaE/40	*a*	APC/ PC/ PE	PCB/ PEB	ApcE×1/ CpcG×1/ CpcL×2	Nostocales/ Calotrichaceae	AP018179, AP018178	WP_096697252/ WP_096598684		WP_06607250	WP_096607253, WP_096605772
Calothrix sp. NIES-3974	CA1/ CA2/ CA6	CcaS + RfpA /12	*a, f*	APC/ PC/ PE	PCB/ PEB	ApcE×2/ CpcG×3/ CpcL×2	Nostocales/ Calotrichaceae	AP018254	WP_096620213/ WP_096625932	WP_096620787, BAZ05880	WP_096620257, WP_096621832, WP_096621834	WP_096625075, WP_096620258
Calothrix sp. NIES-4101	CA1/ CA2	CcaS / 28	*a*	APC/ PC/ PE	PCB/ PEB	ApcE×1/ CpcG×2/ CpcL×2	Nostocales/ Calotrichaceae	AP018279, AP018280, AP018275, AP018277, AP018278, AP018274, AP018276	BAZ37790		BAZ38426, BAZ41692	BAZ38425, BAZ38136
Calothrix sp. PCC 7103	CA1/ CA2	CcaS /29	*a*	APC/ PC/ PE	PCB/ PEB	ApcE×1/ CpcG×2/ CpcL×1	Nostocales/ Calotrichaceae	ALVJ00000000	WP_019489881		WP_019488565, WP_019495782	WP_019495781
Calothrix sp. PCC 7507	CA6	RfpA / 22	*a, d, f*	APC/ PC	PCB	ApcE×2/ CpcG×3/ CpcL×1	Nostocales/ Calotrichaceae	CP003943	WP_015126594	WP_015126587, WP_015126818	WP_015129903, WP_015129904, WP_015129906	WP_015129905
Chamaesiphon minutus PCC 6605	CA3	RcaE / 18	*a*	APC/ PC/ PE	PCB/ PEB	ApcE×1/ CpcG×2/ CpcL×1	Synechococcales/ Chamaesiphonaceae	CP003602, CP003601, CP003600	WP_015161117		WP_015158278, WP_015160460	WP_015160046
Chamaesiphon polymorphus CCALA 037	CA3	RcaE / 15	*a*	APC/ PC/ PE	PCB/ PEB	ApcE×1/ CpcG×2/ CpcL×1	Synechococcales/ Chamaesiphonaceae	PVWO00000000	WP_106310833		WP_106306223, WP_106307793	WP_106312097

Chlorogloeopsis fritschii PCC 6912	CA6	RfpA /25	*a, d, f*	APC/ PC	PCB	ApcE×2/ CpcG×3/ CpcL×1	Nostocales/ Chlorogloeopsidaceae	AJLN00000000	WP_016879258	WP_016873423, WP_016874650	WP_016874681, WP_016874680, WP_016874678	WP_016874679
Chondrocystis sp. NIES-4102	CA3	RcaE /9	*a*	APC/ PC/ PE	PCB/ PEB	ApcE×1/ CpcG×1	Chroococcales/ Chroococcaceae	AP018287, AP018282, AP018284, AP018286, AP018283, AP018285, AP018281	WP_096722896		WP_096722389	
Chroococcidiopsis thermalis PCC 7203	CA6	RfpA /19	*a, d, f*	APC/ PC	PCB	ApcE×2/ CpcG×3	Chroococcidiopsidales/ Chroococcidiopsidaceae	CP003598, CP003597, CP003599	WP_015153109	WP_015153116, WP_015156255	WP_015155417, WP_015155418, WP_015155419	
Fischerella muscicola PCC 7414	CA6	RfpA /29	*a, f*	APC/ PC	PCB	ApcE×2/ CpcG×3/ CpcL×1	Nostocales/ Hapalosiphonaceae	AJLK00000000	WP_102204993	WP_016866291, WP_016868701	WP_016866766, WP_016866768, WP_016866769	WP_016866767
Fischerella sp. NIES-4106	CA6	RfpA /22	*a, f*	APC/ PC	PCB	ApcE×2/ CpcG×3/ CpcL×1	Nostocales/ Hapalosiphonaceae	AP018299, AP018303, AP018298, AP018300, AP018304, AP018302, AP018301, AP018306, AP018305	WP_096680226	WP_096678225, WP_096680239	WP_096677901, WP_096677903, WP_096677904	WP_096677902
Fischerella sp. PCC 9605	CA6	RfpA /26	*a, f*	APC/ PC	PCB	ApcE×2/ CpcG×3/ CpcL×1	Nostocales/ Hapalosiphonaceae	ALVT00000000	WP_026734721	WP_026734727, WP_026731017	WP_026736671, WP_026736672, WP_026736674	WP_026736673
Fischerella thermalis PCC 7521	CA6	RfpA /20	*a, d, f*	APC/ PC	PCB	ApcE×2/ CpcG×3/ CpcL×1	Nostocales/ Hapalosiphonaceae	AJLL00000000	WP_102148434	WP_009453699, WP_009457006	WP_009454953, WP_009454957, WP_016871767	WP_009454955
Fortiea contorta PCC 7126	CA3	RcaE / 16	*a*	APC/ PC/ PE	PCB/ PEB	ApcE×1/ CpcG×1	Nostocales/ Fortieaceae	ANFJ00000000	WP_017653340		WP_017653030	
Fremyella diplosiphon Fd33/ *Tolypothrix sp. PCC 7601*	CA3	RcaE /36	*a*	APC/ PC/ PE	PCB/ PEB	ApcE×1/ CpcG×1/ CpcL×1	Nostocales/ Tolypothrichaceae	AGCR00000000	WP_045871568		WP_045871433	WP_045872077
Geminocystis sp. NIES-3708	CA2	CcaS / 8	*a*	APC/ PC/ PE	PCB/ PEB	ApcE×1/ CpcG×1	Chroococcales/ Chroococcaceae	AP014817, AP014815, AP014819, AP014816, AP014818, AP014820	WP_197671694		WP_066343099	
Gloeobacter violaceus PCC 7421	N.F.	N.F./ 1	*a*	APC/ PC/ PE	PCB/ PEB	ApcE×1	Gloeobacterales/ Gloeobacteraceae	NC_005125				
Halomicronema hongdechloris	CA6	RfpA /9	*a, f*	APC/ PC	PCB	ApcE×2/ CpcG×1	Pseudanabaenales/ Prochlorotrichaceae	CP021983	WP_080806407	WP_080806386, WP_080813471	WP_080808221	
Leptolyngbya boryana PCC 6306	CA1	CcaS /13	*a*	APC/ PC	PCB	ApcE×1/ CpcG×2/ CpcL×1	Pseudanabaenales/ Leptolyngbyaceae	ALVM00000000	WP_017290979		WP_017286918, WP_017288649	WP_026148987
Leptolyngbya sp. Heron Island J	CA3	RcaE /9	*a*	APC/ PC/ PE	PCB/ PEB	ApcE×1/ CpcG×1/ CpcL×2	Pseudanabaenales/ Leptolyngbyaceae	AWNH00000000	WP_023076387		WP_023072014	WP_023072999, WP_023073188
Leptolyngbya sp. NIES-2104	N.F.	N.F./10	*a*	APC/ PC	PCB	ApcE×1/ CpcG×3	Pseudanabaenales/ Leptolyngbyaceae	BBWW00000000			WP_058997656, WP_058997658, WP_058997660	
Leptolyngbya sp. NIES-3755	N.F.	N.F./13	*a*	APC/ PC	PCB	ApcE×1/ CpcG×3	Pseudanabaenales/ Leptolyngbyaceae	AP017308, AP017310, AP017311, AP017309			WP_068390530, WP_068390532, WP_068390535	
Leptolyngbya sp. PCC 6406	CA1	CcaS / 15	*a*	APC/ PC	PCB	ApcE×1/ CpcG×1/ CpcL×2	Pseudanabaenales/ Leptolyngbyaceae	ALVV00000000	WP_008314551		WP_008310042	WP_008314556, WP_008308963

Mastigocoleus testarum BC008	CA3/ CA6	RcaE + RfpA / 40	*a, f*	APC/ PC/ PE	PCB/ PEB	ApcE×3/ CpcG×1/ CpcL×2	Nostocales/ Hapalosiphonaceae	LMTZ00000000	WP_058184871/ WP_036267670	WP_036265308, Ga0125915_110724	WP_027840162	WP_036265220, WP_027840161
Microcystis sp. MC19	CA2	CcaS / 5	*a*	APC/ PC/ PE	PCB/ PEB	ApcE×1/ CpcG×1	Chroococcales/ Microcystaceae	CP020664	WP_106908049		WP_045359953	
Moorea bouillonii PNG5-198	CA3	RcaE /11	*a*	APC/ PC/ PE	PCB/ PEB	ApcE×1/ CpcG×1/ CpcL×1	Oscillatoriales/ Oscillatoriaceae	MKZS00000000	WP_075901034		WP_075904242	WP_075904179
Moorea producenes PAL-8-15-08-1	CA3	RcaE /13	*a*	APC/ PC/ PE	PCB/ PEB	ApcE×1/ CpcG×1/ CpcL×1	Oscillatoriales/ Oscillatoriaceae	CP017708, CP017709, CP017710	WP_070392875		WP_070395606	WP_229424102
Moorena producenes JHB	CA3	RcaE /11	*a*	APC/ PC/ PE	PCB/ PEB	ApcE×1/ CpcG×1/ CpcL×1	Oscillatoriales/ Oscillatoriaceae	CP017708, CP017709, CP017710	WP_071103969		WP_071106845	WP_071106820
Myxosarcina sp. GI1	CA3	RcaE /10	*a*	APC/ PC/ PE	PCB/ PEB	ApcE×1/ CpcG×1	Pleurocapsales/ Hyellaceae	JRFE00000000	WP_036483762		WP_036478911	
Nodosilinea sp. LEGE 07298	CA6	RfpA / 25	*a, f*	APC/ PC	PCB	ApcE×2/ CpcG×3	Pseudanabaenales/ Prochlorotrichaceae	JADEXE00000000	WP_194021995	WP_194021615, WP_194025230	MBE9108339, MBE9110147, WP_194025998	
Nodosilinea sp. P-1105	CA6	RfpA /17	*a, f*	APC/ PC	PCB	ApcE×2/ CpcG×2/ CpcL×2	Pseudanabaenales/ Prochlorotrichaceae	SMDQ00000000	WP_169617661	WP_169613084, WP_169614317	NMF84091, NMF84306	WP_206070731, WP_169617321
Nostoc carneum NIES-2107	CA3	RcaE /44	*a*	APC/ PC/ PE	PCB/ PEB	ApcE×1/ CpcG×2/ CpcL×2	Nostocales/ Nostocaceae	AP018183, AP018181, AP018180, AP018182	BAY29549		BAY32051, BAY28925	BAY32745, BAY32582
Nostoc commune NIES-4072	CA3	RcaE / 21	*a*	APC/ PC/ PE	PCB/ PEB	ApcE×1/ CpcG×2	Nostocales/ Nostocaceae	BDUD00000000	WP_244919223		WP_109012707, WP_109008498	
Nostoc flagelliforme CCNUN1	CA3	RcaE /21	*a*	APC/ PC/ PE	PCB/ PEB	ApcE×1/ CpcG×2	Nostocales/ Nostocaceae	CP024785, CP024793, CP024787, CP024791, CP024789, CP024792, CP024786, CP024790, CP024788	WP_100902110		WP_100899899, WP_100897609	
Nostoc punctiforme PCC 73102	CA1/ CA2	CcaS /29	N.F.	APC/ PC/ PE	PCB/ PEB	ApcE×1/ CpcG×2/ CpcL×1	Nostocales/ Nostocaceae	CP001041, CP001039, CP001040, CP001037, CP001042, CP001038	WP_012410152		WP_012410166, WP_012407513	WP_012410151
Nostoc sp. C052	CA2	CcaS / 37	*a, f*	APC/ PC/ PE	PCB/ PEB	ApcE×1/ CpcG×2	Nostocales/ Nostocaceae	CP040279, CP040275, CP040277, CP040272, CP040278, CP040273, CP040274, CP040280, CP040276	WP_179069187		WP_179068455, WP_179067776	
Nostoc sp. CENA543	CA3	RcaE /22	*a*	APC/ PC/ PE	PCB/ PEB	ApcE×1/ CpcG×1/ CpcL×2	Nostocales/ Nostocaceae	CP023281, CP023282, CP023278, CP023279, CP023280, CP023283	WP_03138107		WP_103138090	WP_103135424, WP_103138091
Nostoc sp. KVJ20	CA2	CcaS /30	*a*	APC/ PC/ PE	PCB/ PEB	ApcE×1/ CpcG×1	Nostocales/ Nostocaceae	LSSA00000000	WP_069073327		WP_069074024	
Nostoc sp. NIES-4103	CA3	RcaE /24	*a*	APC/ PC/ PE	PCB/ PEB	ApcE×1/ CpcG×3	Nostocales/ Nostocaceae	AP018289, AP018288	WP_096557144		WP_096557152, WP_096557136, WP_096557781	
Oscillatoriales sp. JSC-12/*Leptolyngbyaceae cyanobacterium JSC-12*	CA6	RfpA /20	*a, f*	APC/ PC	PCB	ApcE×2/ CpcG×4/ CpcL×1	Pseudanabaenales/ Leptolyngbyaceae	[illegible]JUB00000000, CM00163[illegible]	EKQ66830	EKQ68835, EKQ66842	EKQ68809, EKQ68811, EKQ68812, EKQ67899	EKQ68810

Phormidesmis priestleyi ULC007	N.F.	N.F./9	*a*	APC/ PC	PCB	ApcE×1/ CpcG×3	Pseudanabaenales/ Leptolyngbyaceae	PVWG00000000			WP_073074862, WP_073074863, WP_073074864	
Phormidium tenue FACHB-1052	N.F.	N.F./19	*a*	APC/ PC	PCB	ApcE×1/ CpcG×2/ CpcL×1	Oscillatoriales/ Oscillatoriaceae	JACJQN00000000			WP_073608152, WP_073607311	WP_073606855
Plectonema cf. radiosum LEGE 06105	CA6	RfpA /12	*a, f*	APC/ PC	PCB	ApcE×2/ CpcG×3	Oscillatoriales/ Oscillatoriaceae	JADEWL00000000	WP_193923208	WP_193916309, WP_193923202	WP_193926196, WP_193924451, WP_193924454	
Pleurocapsa sp. PCC 7327	CA6	RfpA /13	*a, f*	APC/ PC/ PE	PCB/ PEB	ApcE×2/ CpcG×1/ CpcL×1	Pleurocapsales/ Hyellaceae	CP003590	WP015143524	WP_015143544, WP_015145932	WP_015143734	WP_015143841
Pleurocapsales cyanobacterium LEGE 10410	CA3/ CA6	RcaE + RfpA / 17	*a, f*	APC/ PC/ PE	PCB/ PEB	ApcE×2/ CpcG×1	Pleurocapsales	JADEXJ00000000	MBE9046724/ MBE9046865	MBE9046795, MBE9046406	MBE9046663	
Pseudanabaena biceps PCC 7429	CA1	CcaS / 15	*a*	APC/ PC	PCB	ApcE×1/ CpcG×2/ CpcL×1	Pseudanabaenales/ Pseudanabaenaceae	ALWB00000000	WP_009629217		WP_009626008, WP_009627925	WP_009629216
Romeria aff. gracilis LEGE 07310	CA6	RfpA / 6	*a, f*	APC/ PC	PCB	ApcE×2/ CpcG×1/ CpcL×2	Pseudanabaenales/ Romeriaceae	JADEXG00000000	WP_193909340	WP_193906269, WP_193909291	MBE9078133	WP_193904477, WP_193906131
Stanieria cyanosphaera PCC 7437	CA3	RcaE / 20	*a*	APC/ PC/ PE	PCB/ PEB	ApcE×1/ CpcG×4	Pleurocapsales/ Dermocarpellaceae	CP003654, CP003657, CP003653, CP003655, CP003656, CP003658	WP_015194148		WP_015194050, WP_015192211, WP_015191542, WP_015192213	
Stanieria sp. NIES-3757	CA3	RcaE /22	*a*	APC/ PC/ PE	PCB/ PEB	ApcE×1/ CpcG×1	Pleurocapsales/ Dermocarpellaceae	AP017375, AP017376	WP_096383955		WP_096388020	
Synechococcus sp. PCC 7335	CA3/ CA6	RcaE and RfpA /15	*a, d, f*	APC/ PC/ PE	PCB/ PEB	ApcE×2/ CpcG×1/ CpcL×1	Synechococcales/ Synechococcaceae	ABRV00000000	WP_006457142/ WP_038015847	WP_006453887, WP_006455341	WP_006454437	WP_006456318
Synechococcus sp. PCC 7336	CA3	RcaE / 8	*a*	APC/ PC/ PE	PCB/ PEB	ApcE×1/ CpcG×1/ CpcL×1	Synechococcales/ Synechococcaceae	CM001776	WP_202951133		WP_017326224	WP_017326223
Synechococcus sp. strain CC9311	CA4	N.F./ 0	*a*	APC/ PC/ PE	PCB/ PEB	ApcE×1/ CpcG×2/ CpcL×1	Synechococcales/ Synechococcaceae	NC_008319			sync_0515, sync_2488	sync_1249
Synechococcus sp. strain CC9902	CA4	N.F./ 0	*a*	APC/ PC/ PE	PCB/ PEB	ApcE×1/ CpcG×2/ CpcL×1	Synechococcales/ Synechococcaceae	NC_007513			Syncc9902_1881, Syncc9902_0399	Syncc9902_1003
Synechocystis sp. PCC 6803	CA1	CcaS /10	*a*	APC/ PC	PCB	ApcE×1/ CpcG×1/ CpcL×1	Synechococcales/ Merismopediaceae	NC_017052.1	WP_014407164		WP_010871747	WP_010874215
Tolypothrix tenuis PCC 7101	CA3	RcaE / 36	*a*	APC/ PC/ PE	PCB/ PEB	ApcE×1/ CpcG×2/ CpcL×2	Nostocales/ Tolypothrichaceae	AP018252, AP018250, AP018253, AP018251, AP018249, AP018248	WP_096577488		WP_096575345, WP_096577410	WP_096574564, WP_096574038
Xenococcus sp. PCC 7305	CA3	RcaE /13	*a*	APC/ PC/ PE	PCB/ PEB	ApcE×21/ CpcG×3/ CpcL×1	Pleurocapsales/ Xenococcaceae	ALVZ00000000	WP_006511570		WP_006511402, WP_006510102, WP_006508305	WP_006510547

N.F. = not found.
*Photoreceptors: CcaS, RcaE and RfpA.

Supplementary Figure S1

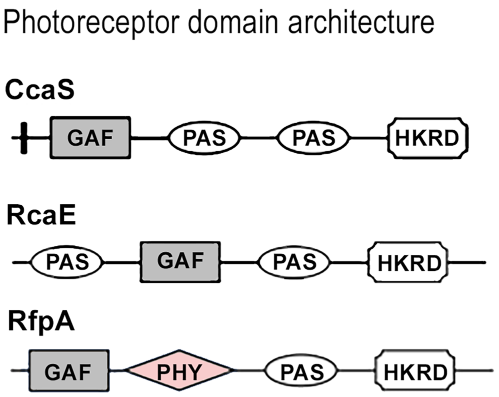

Figure S1. The architecture of known photoreceptors (CcaS, RcaE, and RfpA). Photoreceptors in different species includes a variety of functional domains. Vertical black line represents the transmembrane domains. PHY, phytochrome-like domain; GAF, cGMP phosphodiesterase/adenylate cyclase/FhlA; PAS, Per-Arnt-Sim domain; HKRD, histidine kinase-related domain.

Supplementary Figure S2

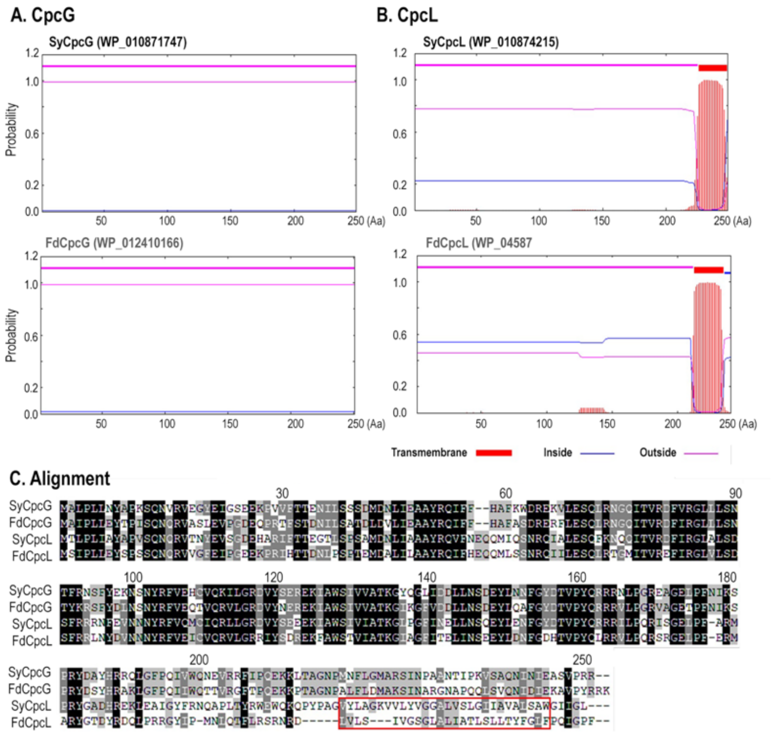

Figure S2. The differences between CpcG and CpcL. A, the predicted secondary protein structure of CpcG proteins using TMHMM program. B, the predicted secondary protein structure of CpcL proteins using TMHMM program. C, The pile-up alignment of CpcG and CpcL proteins. Sy, *Synechocystis* sp. PCC 6803; Fd, *Fremyella diplosiphon* Fd33.

Supplementary Figure S3

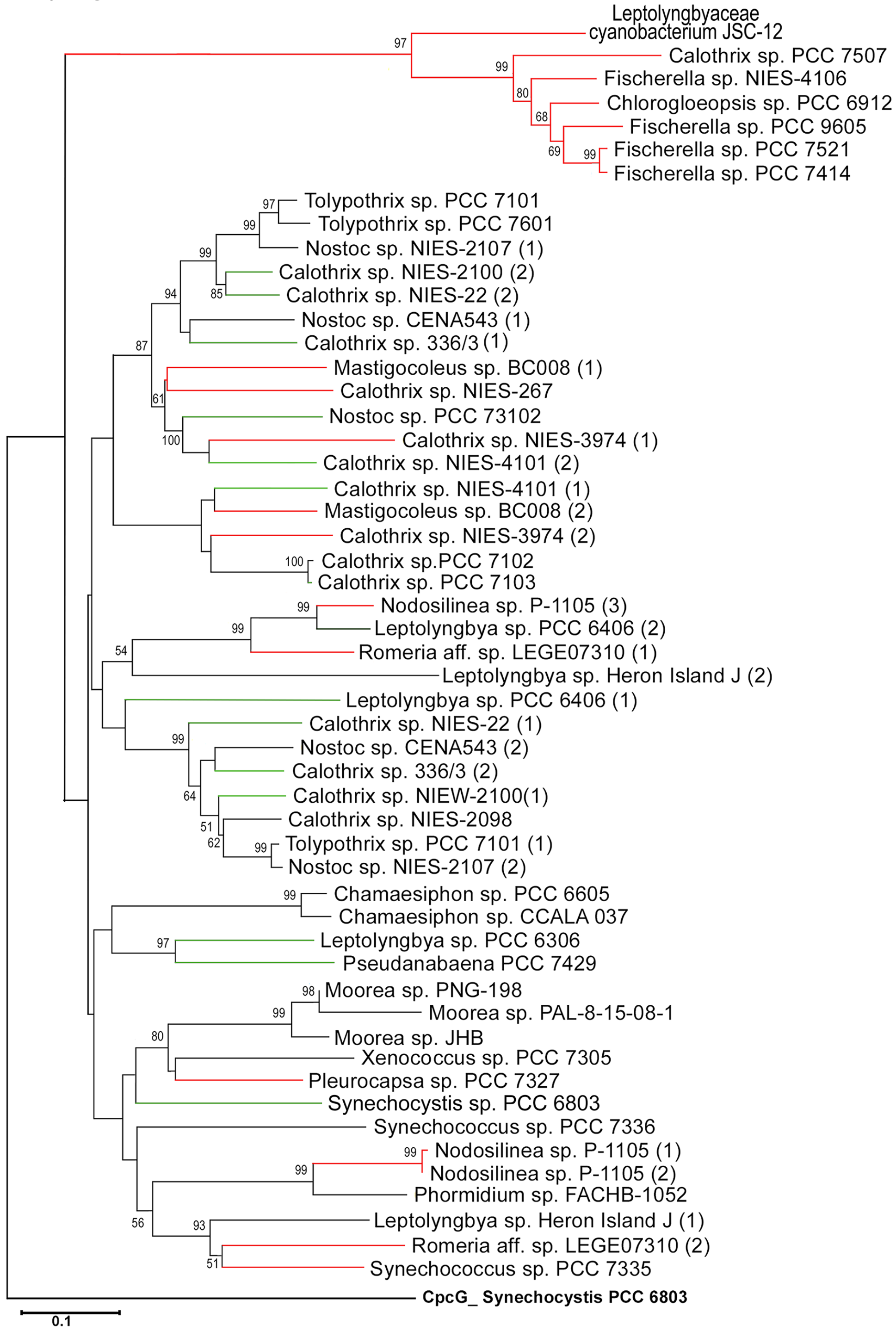

Figure S3. Neighbor-joining phylogeny of CpcL proteins. The number in brackets is used to distinguish multiple copies of CpcL from the same strain. The branches are supported with a bootstrap of ≥50% from 500 replicating calculations. CpcG from *Synechocystis* sp. PCC 6803 is used as outgroup. Green branch lines highlight CpcL from CA1-capable cyanobacteria. Red branch lines highlight CpcL from CA6-capable cyanobacteria. The CpcL sequence accessions are listed in Table S1.
